# Evaluation of propidium monoazide for 16S ribosomal RNA metabarcoding assessment of microbial communities in 60-day ripened raw goat milk cheese

**DOI:** 10.3168/jdsc.2025-0894

**Published:** 2026-01-16

**Authors:** Sintia Naianne Pereira Feitoza, Laiorayne Araújo de Lima, Carla Aparecida Soares Saraiva, Weslla da Silva Dias, Ana Beatriz Azevedo de Medeiros, Artur Cezar de Carvalho Fernandes, Marcos Bryan Heinemann, Fernando Nogueira de Souza, Nivea Regina Oliveira Felisberto, Mateus Lacerda Pereira Lemos, Antônio Silvio do Egito, Celso José Bruno de Oliveira

**Affiliations:** 1 Laboratório de Avaliação de Produtos de Origem Animal (LAPOA), Departamento de Zootecnia (DZ), Centro de Ciências Agrárias (CCA), Universidade Federal da Paraíba (UFPB), Areia, Paraíba, Brasil; 2 Departamento de Medicina Veterinária Preventiva e Saúde Animal, Faculdade de Medicina Veterinária, Universidade de São Paulo (USP), São Paulo-SP, Brasil; 3 Embrapa Caprinos e Ovinos, Campina Grande-PB, Brasil

## Abstract

Cheese ripening is a complex microbial process marked by significant shifts in microbial composition. Considering that propidium monoazide (PMA) depletes DNA from nonviable cells, we hypothesized that PMA treatment of cheese samples could affect the microbiota characterization of 60-d-ripened raw goat curd cheese by 16S rRNA metabarcoding sequencing. After ripening, PMA-treated and nontreated (control) samples from the same cheese units were processed for DNA extraction, library preparation, and 16S rRNA metabarcoding sequencing on an Illumina MiSeq platform. Downstream bioinformatic analyses for microbial diversity assessment were performed using QIIME 2 and the phyloseq package in R. Statistical analyses included permutational multivariate analysis of variance (PERMANOVA), Wilcoxon tests, and linear discriminant analysis effect size (LEfSe). No significant differences were observed in either α or β diversity metrics between PMA-treated and nontreated samples. However, PMA treatment significantly reduced the abundance of farm environment–associated *Dickeya* and *Pectobacteriaceae* taxa in cheese samples, thus improving the accuracy of determining the cheese microbial structure using next-generation sequencing technologies. Further longitudinal studies focusing on different sampling periods during ripening, as well as other cheese types, may shed light on the potential benefits of using PMA for improving the accuracy of cheese microbial community characterization by next-generation sequencing.

## Highlights

• Environmental contaminants in ripened cheese decreased with PMA treatment.
• PMA removes dead cell DNA but does not change overall cheese microbial diversity.
• PMA improves accuracy of viable microbiota detection in ripened goat cheese.

## Graphical Abstract

**Figure:**
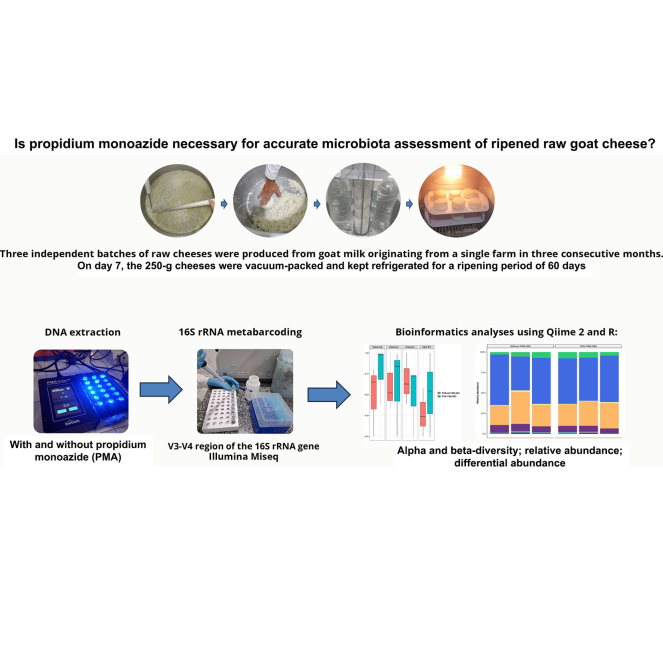
**Summary:** This study evaluated whether propidium monoazide (PMA) treatment affects the characterization of microbial communities in 60-day-ripened raw goat cheese using 16S ribosomal RNA (rRNA) metabarcoding. The PMA selectively depletes DNA from nonviable cells, potentially providing a more accurate representation of living microorganisms. Paired samples (PMA-treated and nontreated controls) from the same cheese units were analyzed using Illumina MiSeq sequencing, with downstream analyses performed in QIIME 2 and R. Results showed no significant differences in alpha or beta diversity metrics between treatments, indicating that PMA did not alter the overall microbial community structure. However, PMA treatment significantly reduced the abundance of farm environment-associated taxa, specifically *Dickeya* and *Pectobacteriaceae*, suggesting these organisms were predominantly nonviable with residual DNA persisting in the cheese matrix. This demonstrates that PMA pretreatment improves the accuracy of cheese microbiome characterization by filtering dead cell DNA from environmental contaminants, providing clearer insights into viable microbial communities during cheese ripening.

Artisanal raw milk cheeses have been gaining increasing recognition worldwide due to their gastronomic value and complex microbial ecosystems. These products are traditionally crafted using artisanal techniques that are deeply influenced by local cultural practices, resulting in distinctive sensory profiles for each cheese variety (Ramírez-Rivera et al., 2017; Ströher et al., 2023). Moreover, raw milk cheeses harbor a rich and diverse natural microbiota, which plays a pivotal role in the development of both desirable and undesirable flavor attributes during ripening (Zheng et al., 2018; Pisano et al., 2019; Nelli et al., 2023).

Although next-generation sequencing (**NGS**) technologies have revolutionized our capacity to characterize and understand the complex microbial structure of different food matrices, a major limitation of DNA-based culture-independent methods is their inability to distinguish between DNA from viable and nonviable cells, potentially leading to misleading conclusions about the active microbiota (Zeng et al., 2016). Propidium monoazide (**PMA**) is a photo-reactive dye that selectively penetrates cells with compromised membranes, such as those of dead microorganisms, preventing DNA amplification (Qin et al., 2020). It has been used as an alternative approach to deplete information related to nonviable organisms in NGS (Erkus et al., 2016). Considering the important microbial shifts that occur during ripening, which could lead to the presence of nonviable bacteria, our rationale is that PMA use could result in more accurate microbial profiling using NGS approaches.

This study was designed to test the null hypothesis (*H*_0_) that there are no differences in the microbial diversity of PMA-treated and nontreated raw goat curd cheese samples at 60 d of ripening.

The experiment was conducted using a completely randomized design with 2 treatments (PMA-treated and nontreated samples) and 3 replicates (cheese units). The 3 cheese units (∼250 g each) were produced from a single raw goat milk batch, ripened under controlled conditions, and sampled at 60 d. Two aliquots from each replicate were randomly assigned to one of 2 treatments: with or without PMA. Therefore, DNA libraries were prepared from 3 individual samples (biological replicates) from each treatment and sequenced.

Raw goat milk (15 L) was obtained from a small-scale commercial goat farm in Puxinanã, Paraíba, Brazil, immediately after milking and transferred into three 5-L aluminum milk buckets, which had been previously sanitized using a 2.5% sodium hypochlorite solution. The buckets were immediately placed in insulated boxes with ice and transported to the Dairy Laboratory of the Federal University of Paraíba (CCA/UFPB). The raw milk was filtered and transferred to a stainless-steel vat, and heated to 35°C. At this temperature, 400 μL of rennet (Vilac Foods, RN, Brazil) was added. After thorough mixing, the milk was left undisturbed for 45 min to allow coagulation. The curd was then cut into 1- to 2-cm cubes and gently stirred for 30 min without rest. The whey was drained, and 45 g of salt was added directly to the curd. After homogenization, the cheese curd was transferred into round cheese molds (approximately 250 g each). During pressing, the cheeses were turned for the first time after 20 min, and again 90 min later. The pressing process continued for a total of 4 h.

After pressing, the cheese was kept in the molds and stored in the refrigerator overnight. The cheese remained covered with a clean, damp cloth for 6 d. On the seventh day, the cheeses were vacuum-packed in transparent plastic bags and kept refrigerated for a ripening period of 60 d, which is the minimum ripening time established by Brazilian legislation for raw milk cheeses (Brasil, 1996).

Complete slices (approximately 10 g), including both rind and crumb portions, were taken from 2 opposite quadrants of each cheese using sterilized utensils, transferred into sterile stainless-steel dishes, and macerated using a sterile spatula. For fat removal, we followed a previously published protocol (Quigley et al., 2012). Briefly, sample aliquots (0.250 ± 0.05 g) were transferred into sterile microtubes containing 250 µL of Dulbecco's phosphate-buffered saline (**DPBS**), homogenized using a sterile spatula, and vortexed. The supernatant was discharged, and the resulting pellet was resuspended in 250 µL of DPBS and vortexed. Subsequently, 250 μL of isoamyl alcohol was added to each sample, followed by incubation at 45°C for 10 min and centrifugation at 14,000 × *g* for 10 min at 4°C. After discarding the supernatant, the pellets were incubated at 37°C for 10 min, washed twice with DPBS, and then stored at −80°C for subsequent analyses.

A 25-μL aliquot of a 20 μmol/L PMA solution (Biotium, Fremont, CA) was added to the pellet obtained after fat removal, which was then incubated on ice in the dark for 20 min, followed by light exposure for 15 min using a photolysis device (PMA-Lite LED Photolysis Device, Biotium). Subsequently, the samples were centrifuged at 14,000 × *g* for 10 min at 4°C to pellet the bacterial cells. The resulting pellets were stored at −20°C for subsequent 16S rRNA gene sequencing. The DNA extraction from cheese samples was performed using a commercial kit (DNeasy PowerSoil Pro Kit, Qiagen, USA), following the manufacturer's guidelines. The DNA quantification and integrity were assessed by fluorometric assay (Qubit 3.0, Life Technologies, Carlsbad, CA) and by agarose gel electrophoresis, respectively.

The V3–V4 hypervariable regions of the 16S rRNA gene were targeted for sequencing, according to a reference protocol (Illumina, 2013). The resulting sequences were demultiplexed into FASTQ files, and downstream analysis was performed using QIIME 2 (Bolyen et al., 2019). Low-quality reads were filtered, and chimera removal was performed using USEARCH 6.1. Sequences were clustered into amplicon sequence variants (**ASV**) at 98% similarity and aligned against the SILVA SSU reference database (Quast et al., 2013).

Alpha diversity was assessed using the following metrics: observed ASV, Shannon index, Simpson index, and Faith's phylogenetic diversity (**Faith PD**). For β diversity, both weighted and unweighted UniFrac distance matrices were calculated (Lozupone and Knight, 2008). Principal coordinate analysis (**PCoA**) visualizations were generated using the Emperor tool. Output files from the QIIME 2 analysis pipeline were further processed in R using the phyloseq package v.1.20.0 (McMurdie and Holmes, 2013), and bar plots were created using the ggplot2 package (v.2.2.1, Wickham, 2026) in RStudio (v.1.0.143, RStudio Team, 2019).

Analysis of molecular variance (permutational multivariate analysis of variance; **PERMANOVA**) for assessing differences in taxa richness and evenness (α diversity) among samples was performed using GraphPad Prism software (GraphPad v.5.0, San Diego, CA). The Wilcoxon signed-rank test was applied to compare medians between paired datasets. Parsimony tests were conducted to identify evolutionary differences among samples, and the resulting data were used to construct dendrograms representing evolutionary distances. Differential analysis was performed using linear discriminant analysis effect size (**LEfSe**) to evaluate the relative abundance of ASV across the different experimental groups.

Sequencing yielded 78,191 raw reads in total, with an average of 13,031.83 reads per sample. For α and β diversity assessment, a rarefaction depth of 3,906 reads per sample was applied, with no loss of microbial diversity as confirmed by the rarefaction curves. After rarefaction to 3,906 reads, the normalized dataset comprised 23,436 reads. A total of 175 ASV were identified. The α diversity metrics (observed ASV, Shannon, Simpson, and Faith PD) are presented in Figure 1. The PMA treatment at d 60 did not significantly affect any of the diversity indices evaluated. For β diversity, PCoA based on both unweighted and weighted UniFrac showed high intersample variability with scattered sample distribution across the ordination space, regardless of treatment (Figure 2). The PERMANOVA confirmed no significant treatment effect on overall community structure.

**Figure 1:**
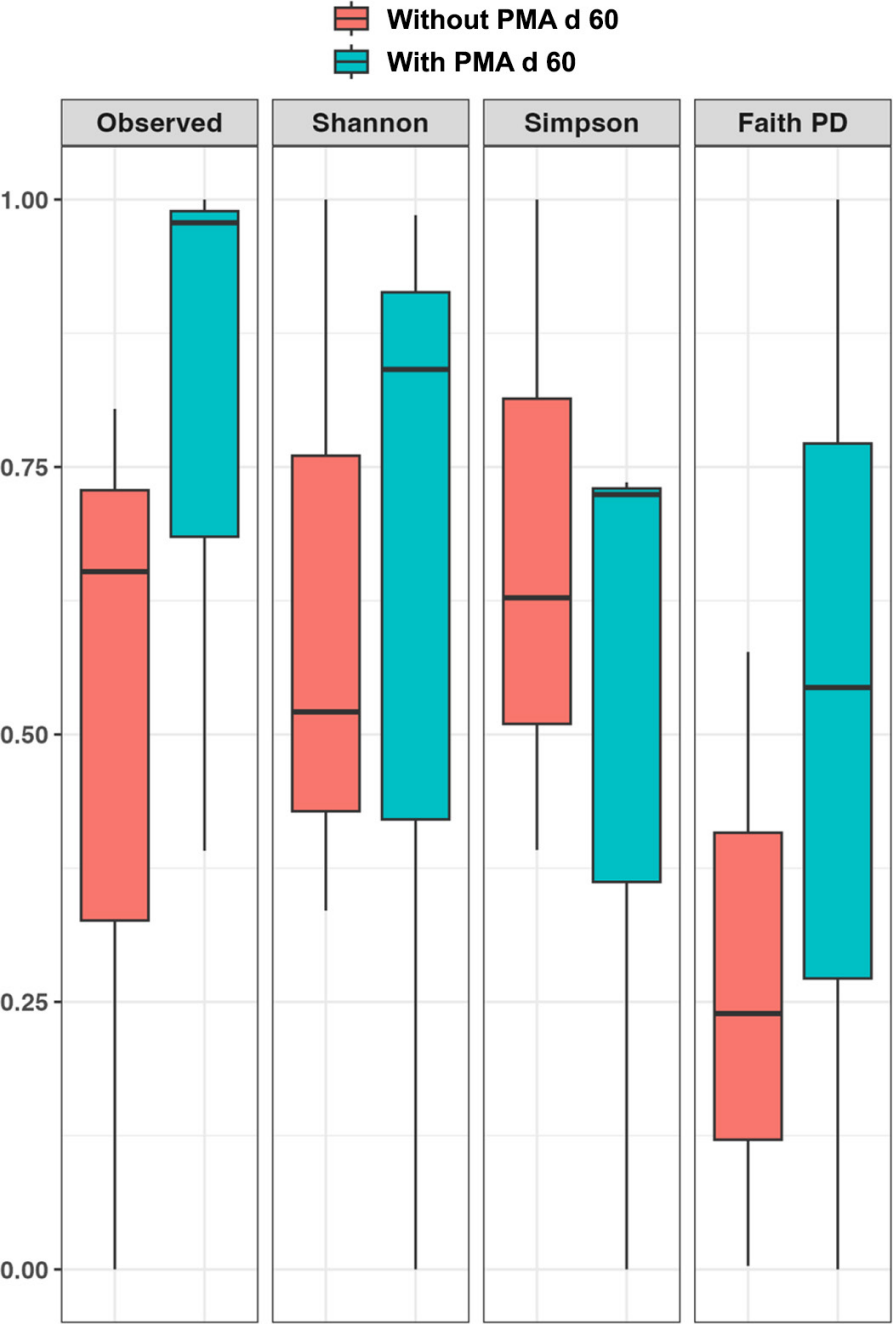
Alpha diversity metrics of microbial communities in 60-d-ripened raw goat cheese samples with and without propidium monoazide (PMA) treatment. Diversity was assessed using 4 complementary metrics: observed amplicon sequence variants (ASV), Shannon diversity index, Simpson diversity index, and Faith's phylogenetic diversity (Faith PD) index. The box represents the interquartile range containing the middle 50% of the data, the line inside the box marks the median value, and the whiskers extending from the box indicate the minimum and maximum observed values.

**Figure 2:**
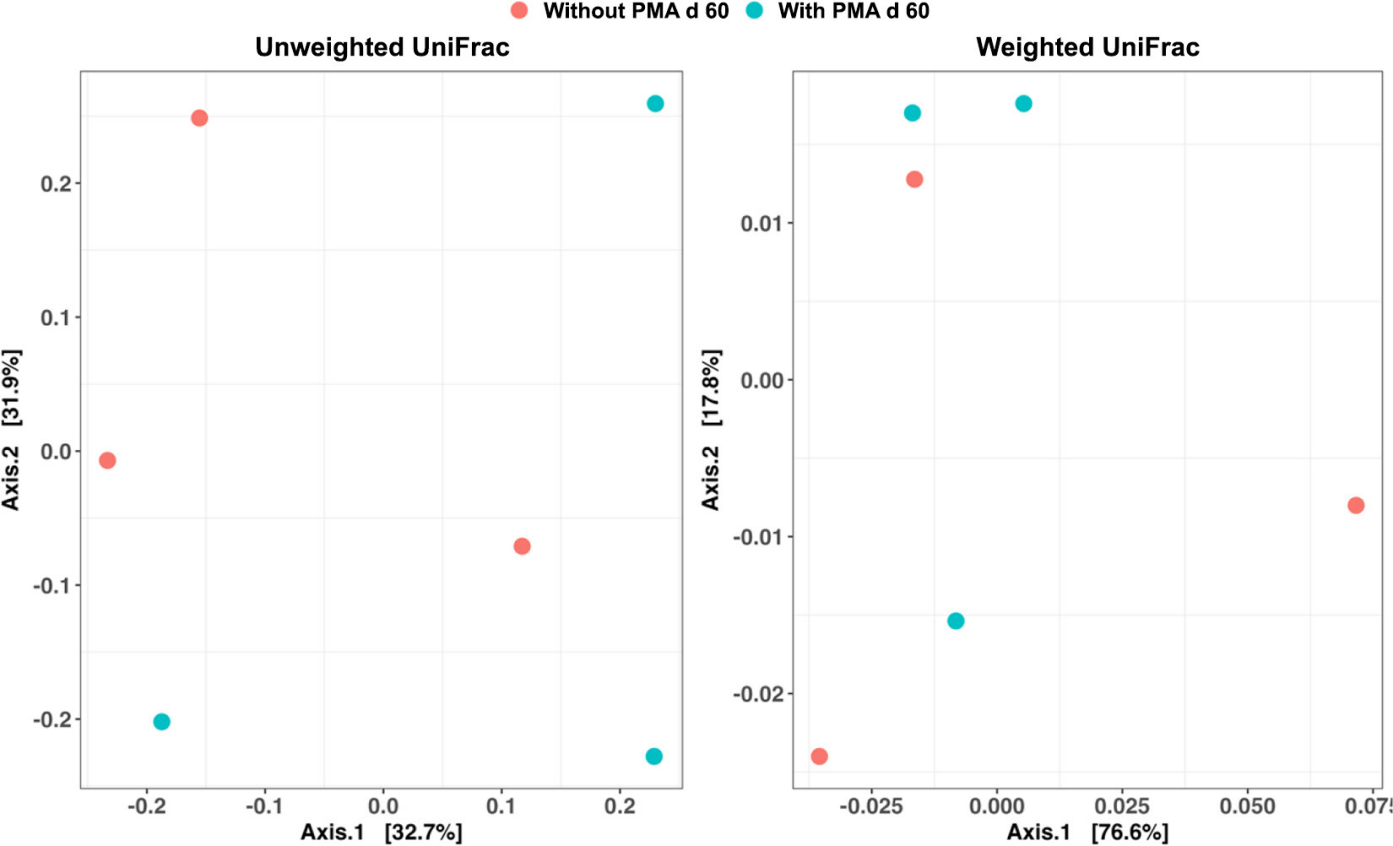
Principal coordinate analysis based on both unweighted and weighted UniFrac dissimilarity matrices of microbial communities in 60-d-ripened raw goat cheese samples with and without propidium monoazide (PMA) treatment.

Barzideh et al. (2022) were able to detect an early decrease in *Lactococcus* (around 1 mo) in PMA-treated compared with total DNA samples during long-term ripening of Cheddar cheese. Importantly, their results from qPCR revealed that the highest difference (1.5 to 2.5 log) in *Lactococcus* spp. quantification between PMA-treated and total DNA occurred from 3 to 6 mo of ripening, which is longer than the ripening time used in our study. Propidium monoazide was also successfully used to improve the accuracy of viable cell quantification for both *Lactococcus* and *Leuconostoc* starter cultures used in bovine cheese production (Erkus et al., 2016). These studies used PMA in a less complex matrix (pasteurized milk inoculated with starter cultures) compared with raw cheese. This may be a critical factor because PMA has limited effects in improving the quantitative assessment of taxa in complex communities (Wang et al., 2021). This observation is particularly relevant for cheese microbiomes derived from raw milk, which represent highly complex ecosystems with intricate microbial interactions. Therefore, different cheese types, environmental conditions, production methods, and ripening periods may explain the contrasting results. Cheese-specific physicochemical intrinsic factors, such as high salt content and low pH (typical of aged cheeses), may also directly affect PMA penetration and effectiveness. The water activity and ionic strength of the cheese matrix may further influence PMA distribution and photo-activation efficiency. Moreover, although we employed a specific protocol to reduce fat and protein contents in the samples, it is possible that residual concentrations of these components interfered with PMA effectiveness. The presence of cheese proteins, particularly casein-derived peptides, and lipid droplets may create physical barriers or binding sites that compete with PMA for cellular targets, potentially reducing the selective depletion of DNA from nonviable cells.

The microbial structural compositions of the 60-d-ripened raw goat cheese samples with and without PMA treatment at family and genus taxonomic levels are shown in Figure 3. Very similar compositions were observed across samples from both treatments, which were composed mainly of *Lactococcus* spp. and *Leuconostoc* spp. organisms. The presence of *Pseudomonas* spp. in both treatments warrants monitoring during extended ripening periods, as this psychrotrophic genus is commonly associated with refrigerated cheese and can produce proteolytic and lipolytic enzymes that may affect sensory attributes, which remain to be further evaluated.

**Figure 3:**
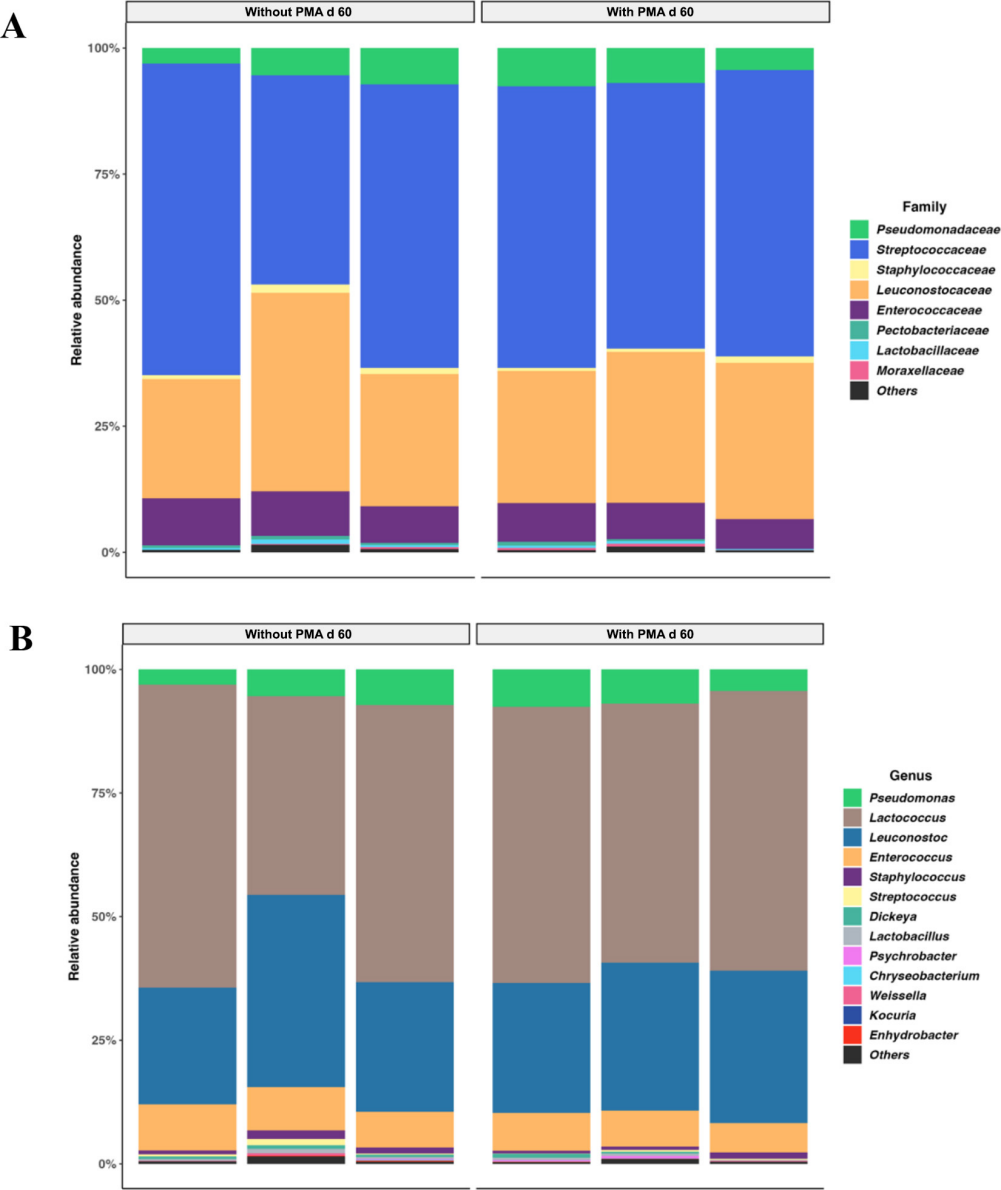
Relative abundance of microbial taxa at the family (A) and genus (B) levels in 60-d-ripened raw goat cheese samples with and without propidium monoazide (PMA) treatment. Data represent the taxonomic composition of bacterial communities based on 16S rRNA gene amplicon sequencing.

Importantly, differential abundance analysis revealed that ASV associated with *Dickeya* and *Pectobacteriaceae* were more abundant in samples without PMA treatment, indicating that these taxa likely represented nonviable cells by the end of the ripening period. *Dickeya* species are primarily plant pathogens adapted to plant tissue environments and are not typically associated with dairy fermentation processes or the natural milk microbiome (Hugouvieux-Cotte-Pattat et al., 2023). Their presence in cheese samples most likely resulted from environmental contamination during milking, processing, or storage, as suggested by their selective depletion following PMA treatment. Therefore, PMA treatment improved the accuracy of the 16S rRNA metabarcoding characterization of 60-d raw goat curd cheese.

The selective action of PMA observed in our study, primarily affecting environmental contaminants, suggests that PMA could be particularly useful in studies focusing on contamination of milk and cheese by environmental organisms. However, the practical importance of this selective depletion in the context of the overall microbial composition of 60-d-ripened raw goat cheese in our study was limited because these low-abundant environmental taxa (*Dickeya* and *Pectobacteriaceae*) do not belong to the core lactic bacteria and do not contribute to cheese ripening dynamics.

From a practical standpoint, the cost-benefit analysis for implementing PMA treatment in studies focusing on the general aspects of raw cheese microbial structure appears unfavorable. Propidium monoazide is a relatively expensive reagent that requires specialized equipment for photo-activation. Given the minimal improvements observed in microbial community characterization, particularly for cheese-associated microbiota, conventional DNA extraction and 16S rRNA sequencing approaches may be more cost-effective for routine cheese microbiome analysis. However, PMA treatment may still be valuable for studies investigating the temporal dynamics of ripening, particularly in cheese types produced with starter cultures. In such studies, the use of PMA could provide insights into microbial succession that are more pronounced in the early stages of ripening. Additionally, PMA could also be useful in studies involving environmental contaminants that may affect cheese safety, such as in studying cheese spoilage scenarios where large numbers of injured or dead cells are present, or in studies using NGS to investigate the effects of surface contact decontamination processes in cheese production environments.

In conclusion, our findings do not provide evidence to reject the null hypothesis (*H*_0_); that is, there are no significant differences in 16S rRNA metabarcoding microbial profiling between PMA-treated and nontreated samples after 60 d of ripening, suggesting that standard microbiome analysis protocols may be sufficient for most applications addressing cheese microbiota characterization by NGS. However, PMA treatment effectively removed DNA from certain environmental contaminants, providing a more accurate characterization of the raw milk cheese microbiota. These aspects should be considered when designing studies addressing NGS-oriented cheese microbial structural compositions. Further longitudinal studies focusing on different sampling periods during ripening, as well as other cheese types, may shed light on the potential benefits of using PMA for improving the accuracy of raw cheese microbial community characterization by NGS. Last, technical optimization of PMA protocols for specific cheese types may be necessary, considering that standardized approaches developed for liquid matrices may not be directly applicable to complex fermented dairy products.
